# The Role of Virtual Reality in the Management of Irritable Bowel Syndrome

**DOI:** 10.1007/s11894-024-00940-w

**Published:** 2024-08-13

**Authors:** Karisma K. Suchak, Christopher V. Almario, Omer Liran, Robert Chernoff, Brennan R. Spiegel

**Affiliations:** 1 Division of Health Sciences Research, Department of Medicine, Cedars-Sinai Health System, Pacific Theatre Building 116 N. Robertson Blvd, Suite 800, Los Angeles, CA 90048 USA; 2 Cedars-Sinai Center for Outcomes Research and Education (CS-CORE), Los Angeles, CA USA; 3 Karsh Division of Gastroenterology and Hepatology, Cedars-Sinai, Los Angeles, CA USA; 4 Cedars-Sinai Department of Psychiatry and Behavioral Sciences, Los Angeles, CA USA

**Keywords:** Irritable bowel syndrome, Virtual reality, Cognitive behavioral therapy

## Abstract

**Purpose of Review:**

Irritable bowel syndrome (IBS) is a disorder of gut-brain interaction that significantly impacts health-related quality of life (HRQOL). This article explores the potential role of virtual reality (VR)-based cognitive behavioral therapy (CBT) in treating patients with IBS.

**Recent Findings:**

While CBT is a proven, skills-based therapy approach that modifies behaviors and alters dysfunctional thinking patterns to influence the gut-brain axis and improve IBS symptoms, it is rarely prescribed given a paucity of CBT-trained clinicians. We developed a novel VR program that delivers a standardized CBT program over an 8-week period to help patients manage their symptoms. In initial qualitative validation testing, patients expressed positive perceptions about using VR CBT for IBS.

**Summary:**

Home-based, standardized VR CBT has the potential to be an effective and scalable treatment option for patients with IBS. While initial studies have shown proof-of-concept definitive randomized controlled trials are needed to demonstrate the efficacy of self-administered VR CBT in IBS.

## Introduction

Irritable bowel syndrome (IBS) is a disorder of gut-brain interaction (DGBI) that is characterized by chronic, recurrent abdominal pain and abnormal bowel habits [1]. IBS significantly impacts biopsychosocial functioning, and individuals diagnosed with IBS experience substantial health-related quality of life (HRQOL) decrements similar to those with diabetes, end-stage renal disease, and congestive heart failure [2–4]. Due to the high disease prevalence and marked impact on HRQOL, the economic burden and healthcare utilization related to IBS are enormous; 2.3 M outpatient visits each year are due to IBS [5–7]. 

While there are many medications used to treat IBS (e.g., neuromodulators, secretagogues, antibiotics) [8], medical therapy often falls short in delivering meaningful, sustained improvements in symptoms and HRQOL. The average number needed to treat (NNT) for US Food and Drug Administration (FDA)-approved IBS medications is 9 [9]. Many of the therapies also have significant side effects that lead patients to discontinue therapy. For example, patients are commonly prescribed tricyclic antidepressants which can cause somnolence, severe dry mouth, and constipation, among other side effects. Hence, there is a critical gap in managing IBS; it is vital to develop and test novel behavioral strategies. In this article, we discuss the evolving role of virtual reality (VR) as a treatment option for IBS, and focus on a novel, on-demand virtual reality VR-based cognitive behavioral therapy (CBT) program that our team developed for patients with IBS.

## CBT in IBS

In the biopsychosocial model of IBS pathogenesis, there are interactions between biology (e.g., genetic predisposition, intestinal dysmotility, gut microbiome changes), behavior (e.g., illness behavior, symptom avoidance), cognitive processes (e.g., brain-gut dysregulation, visceral anxiety), and environment (e.g., childhood trauma, stress) [4, 10]. Dysregulation in any of these factors can lead to IBS symptoms, particularly alterations in the gut-brain axis; central nervous system alterations (e.g., chronic stress, anxiety, depression) can lead to symptoms via changes in tight junction and intestinal permeability that cause inflammation, edema, and changes in visceral neuromuscular function [8, 11, 12]. The above findings combined with the well-established overlap of IBS with psychological comorbidities, coupled with evidence that neuromodulators and psychotherapies are effective, indicate that IBS is a disorder of gut-brain interaction [13–16]. 

Cognitive processes such visceral hypersensitivity and visceral anxiety underlie symptoms for many patients with IBS [10, 12]. CBT is a skills-based therapy approach that targets these cognitive processes by modifying behaviors and altering dysfunctional thinking patterns to influence the gut-brain axis and improve IBS symptoms [10, 17, 18]. Moreover, CBT has been well-studied in IBS with greater than 30 randomized clinical trials (RCTs) demonstrating its efficacy [13–15, 17, 19–21]. For example, Lackner et al. performed a pioneering RCT comparing standard CBT (10 clinic sessions covering: brain-gut interactions; monitoring of symptoms, triggers, and consequences; muscle relaxation; problem solving; relapse prevention training) vs. home-based CBT with minimal therapist contact (4 therapist sessions and home materials on the same topics as standard CBT) vs. IBS education (4 support and informational sessions about IBS and lifestyle factors) [19]. At week 12, more people had global improvement in IBS symptoms in both the standard CBT (55%; *p* < 0.05) and home-based CBT with minimal therapist contact (61%; *p* < 0.01) arms vs. the education arm (44%; ref) [19]. They also found that the effects were durable at 1 year [22]. 

These data led the American College of Gastroenterology (ACG) and American Gastroenterological Association (AGA) in their IBS clinical guidelines to suggest use of CBT in conjunction with other treatments, as it is low risk and efficacious with a NNT of 4 [9, 15, 23]. Yet, despite the strength of evidence showing its efficacy and endorsement in national guidelines, CBT is rarely prescribed given a paucity of clinicians trained in behavioral techniques; self-administered options are needed to bridge the growing gap between supply and demand of CBT practitioners for IBS and other DGBI. On-demand, standardized CBT programs delivered via VR at home has the potential to bridge this gap.

## VR in IBS and Hypothesized Mechanisms

VR is unlike other audiovisual technologies in its ability to generate meaningful emotional experiences and durable clinical responses [24, 25]. Users of VR wear a head-mounted display that creates a vivid perception of being transported into immersive and emotionally evocative worlds. The effect of VR is mediated through several mechanisms, most notably presence—the ability of VR to convey a compelling sense of “just being there,” wherever *there* happens to be [26, 27]. For example, VR can simulate relaxing on a beach, flying over nature scenes, or swimming with dolphins, among countless other environments. Research has shown that these virtual journeys can durably impact clinical outcomes even 18–24 months post treatment [25, 28]. 

In the context of IBS, VR has the potential to administer an on-demand, standardized CBT program to teach patients skills to manage the maladaptive cognitive processes (e.g., visceral hypersensitivity, visceral anxiety) contributing to their brain-gut axis symptoms. Over time, patients may employ the new skills they learned in VR in everyday life without requiring ongoing VR exposure, similar to the long-term benefits achieved for somatic pain up to 24 months after discontinuing VR CBT therapy [28]. Simultaneously, use of VR as the delivery mechanism for CBT may also lead to “virtual analgesia” for patients’ abdominal pain—the principal driver of IBS symptom severity and the cornerstone of the IBS illness experience [29–33]. Notably, therapeutic VR has long been used to manage the cognitive, affective, and sensory aspects of pain across diverse conditions [34–45]. By stimulating the visual cortex in a way that standard two-dimensional (2D) apps are unable to achieve, VR distracts users from processing nociceptive stimuli that do not require continued use of VR once cognitive skills are transferred [25]. Studies and meta-analyses reveal that VR helps manage chronic pain conditions such as complex regional pain syndrome, chronic neck pain, chronic lower back pain, and cancer pain, among other conditions [37, 40, 42, 46–49]. 

Our team at Cedars-Sinai has employed virtual analgesia in over 3,000 patients and demonstrated that it is feasible and practical for patients to use the equipment [50]. For instance, we performed a non-randomized trial in 2017 testing VR analgesia in 100 participants with diverse forms of pain; the study demonstrated a 24% reduction in pain that outperformed a 2D relaxation video [51]. Moreover, 65% of VR participants achieved a clinically significant decrease in pain vs. 40% of controls (*p* = 0.01; NNT = 4) [51]. In a follow-up RCT published in 2019, we found additional evidence supporting the analgesic benefits of VR across a wide range of pain conditions (*N* = 120) [52]. This study found that VR was effective across ages [52], and from an ongoing NIH HEAL study we are observing similar VR acceptability and effectiveness by gender, race, and ethnicity.

In short, the use of virtual analgesia has gained traction on the strength of less expensive, more scalable, and higher quality VR equipment, meaningful advances in the science of therapeutic VR, dissemination of methodological guidance for VR trials [53], and patient acceptability of VR.

## Central Nervous System Effects of VR on IBS Abdominal Pain

Although IBS is a multi-symptom disorder, abdominal pain is its defining characteristic and a predominant feature of the IBS illness experience [29–33]. Unlike other IBS symptoms, such as bloating or bowel habit changes, research from our team found that abdominal pain independently drives HRQOL decrements in IBS [32] and is the principal driver of patient-reported symptom severity [31, 33]. In a study of 755 patients with IBS, we discovered that abdominal pain was the strongest predictor of IBS severity among over 25 tested clinical factors [31]. 

**Fig. 1:**
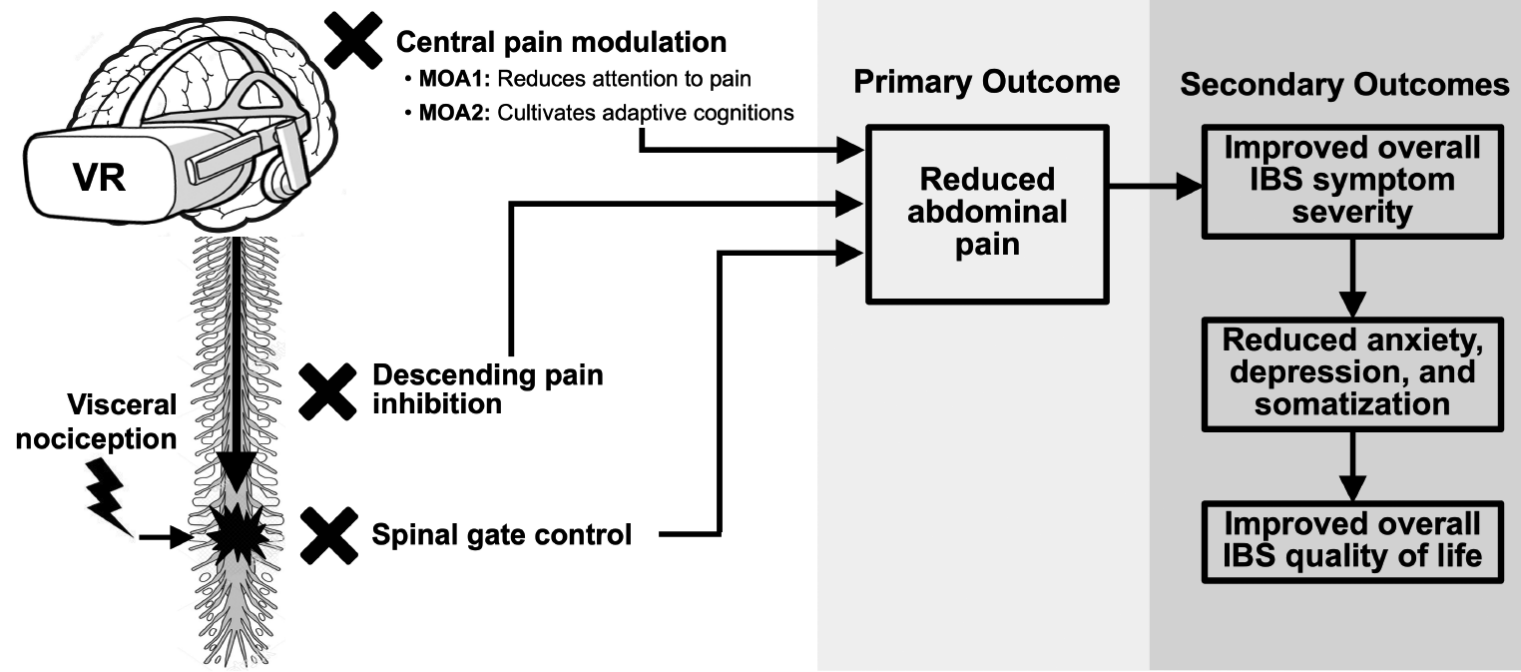
Conceptual model of VR analgesia depicting MOAs and their proposed primary and secondary effects in IBS

Research indicates that VR reduces pain through 2 foundational mechanisms of action (MOAs), herein labeled MOA1 and MOA2 (Fig. 1). First, by stimulating the visual cortex while engaging other senses (e.g., auditory, proprioceptive), VR acts as a distraction to limit the user’s processing of nociceptive stimuli (MOA1) [54]. The result is a form of “inattentional blindness” where attentional bandwidth is redirected to the virtual world, lessening one’s ability to attend to pain signals outside the “spotlight of attention.” [55] By overwhelming the visual, auditory, and vestibular senses, VR creates an immersive distraction that restricts the brain from processing pain. The ability of VR to occupy attention also creates an illusion of *time acceleration*, thus shortening the perception of pain episodes [56–58]. For example, RCTs reveal that VR reduces the perceived length of labor during childbirth, episiotomy repair, endoscopic procedures, and chemotherapy infusions by an average of 30–50% [56–58]. In addition to modifying attention to pain through distraction and time acceleration, VR offers an immersive platform through which people acquire and begin to master specific skills and cultivate adaptive cognitive patterns that reduce pain processing in the central nervous system (MOA2) [59, 60]. 

The net effect of MOA1 and MOA2 is to centrally modulate pain, promote positive affect, and downregulate physical and emotional components of pain. These effects, in turn, leverage the gate control theory of Melzack and Wall [61], wherein VR activates descending inhibitory pathways and dampens spinal transmission of peripheral afferent pain signals [62–64]. Further, distraction-based VR affects pain processing in the sensory and insular cortex, indicating it can reduce both the intensity of pain and the emotional response to pain [65, 66]. Moreover, data indicates that VR produces similar functional MRI effects as hydromorphone and is as effective as opioids at alleviating pain [64]. 

Taken together, the conceptual framework in Fig. 1 suggests that VR may reduce IBS pain via central pain modulation coupled with diminished spinal transmission of pain signals. Based on data that VR offers comparable or adjunctive analgesia to opioids [65], combined with robust RCT data showing that non-VR CBT is effective for IBS [13–15, 19], we hypothesize that using VR-administered CBT may reduce abdominal pain, which will then lead to improved overall physical, psychological, and social functioning. As chronic and recurrent abdominal pain is a defining characteristic and predominant feature of the IBS experience [29–33], use of VR to deliver CBT may lead to incremental benefits for IBS associated abdominal pain over that achieved with traditional CBT alone.

## Enteric Nervous System Effects of VR on IBS Abdominal Pain

Although VR was initially thought to mainly address pain through central nervous system effects, it is now known that there are reciprocal interactions between the central and enteric nervous systems. This is supported by a study from Schneider et al. that demonstrated how the enteric nervous system acts as a relay between psychological stress and gut inflammation and dysmotility [11]. The investigators found that chronically elevated levels of glucocorticoids drive the generation of an inflammatory subset of enteric glia that promotes intestinal inflammation [11]. Glucocorticoids also cause transcriptional immaturity in enteric neurons, acetylcholine deficiency, and subsequently GI dysmotility [11]. By reducing stress and anxiety—both of which are highly prevalent in people with IBS—through VR CBT, a reduction intestinal inflammation and dysmotility may occur, thus leading to improvements in abdominal pain and HRQOL.

## A Novel, On-Demand, VR-Based CBT Program for IBS

### Development

Using the ORBIT Model for Developing Behavioral Treatments for Chronic Diseases framework, our Cedars-Sinai team developed a VR program that delivers CBT over an 8-week period to help patients manage their gut symptoms [67]. As part of the ORBIT Model Phase I (Design), the program was defined and refined by a multidisciplinary team (CBT psychologist, gastroenterologist, psychiatrist, biomedical visualization specialist, VR programmer and developer, human-centered design expert, and digital health researchers) in partnership with patients [68].

**Fig. 2:**
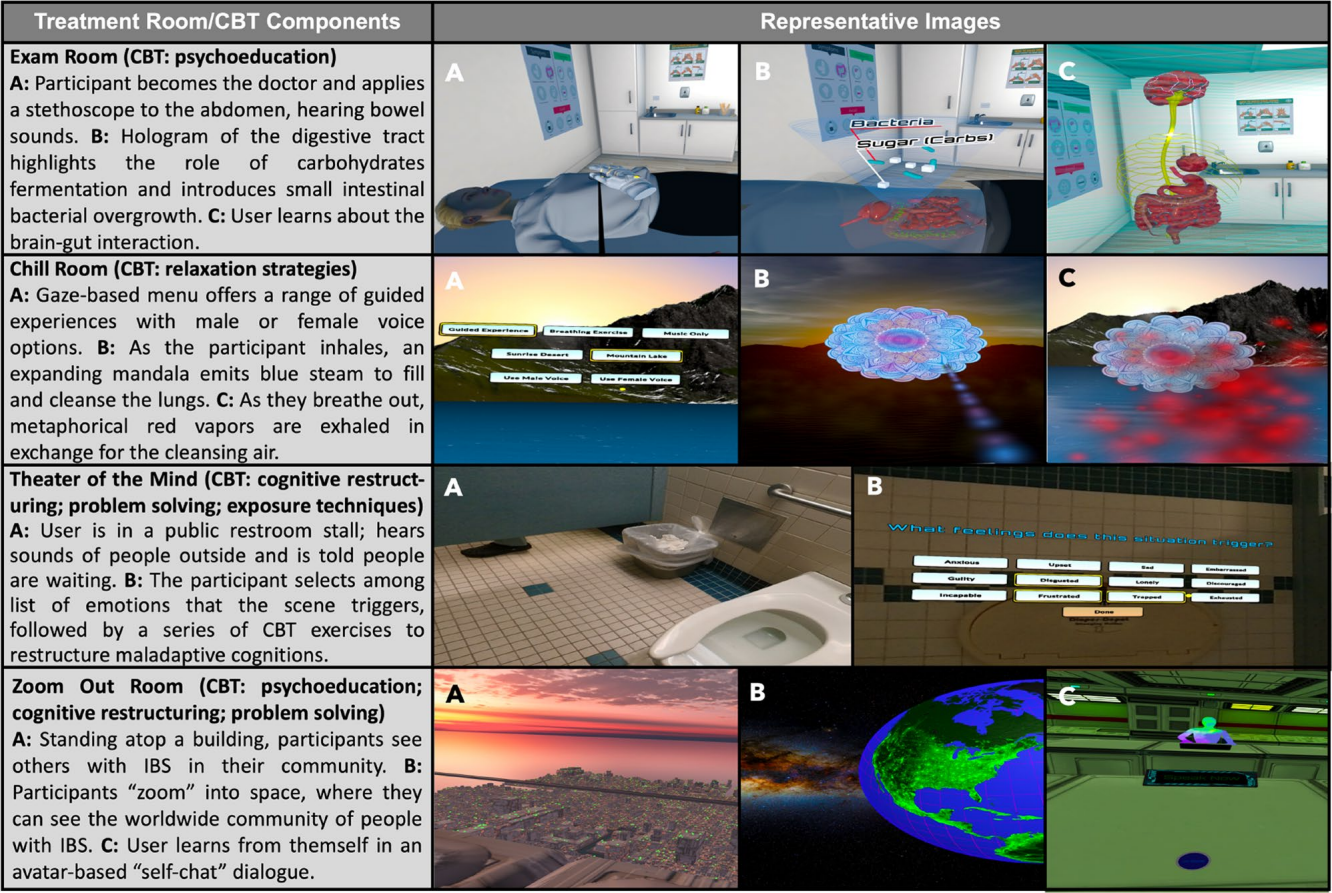
Select images from VR treatment rooms and their CBT components

While actual techniques and foci can vary between CBT frameworks, the VR program includes the following essential CBT components as described by Kinsinger [10]: (i) psychoeducation; (ii) relaxation strategies; (iii) cognitive restructuring; (iv) problem-solving skills; and (v) exposure techniques. Each component maps to 4 VR environments (i.e., “treatment rooms”) that users encounter within the program: (i) Exam Room → psychoeducation; (ii) Chill Room → relaxation strategies and talk therapy; (iii) Theater of the Mind → cognitive restructuring; problem-solving skills; exposure techniques; (iv) Zoom Out Room → psychoeducation; cognitive restructuring; problem-solving skills. Figure 2 shows representative images from each VR treatment room. To optimize accessibility across educational levels, the voiceover scripts were written at an elementary school level with an average Flesch-Kincaid Grade Level score of 6 and Simple Measure Gobbledygook (SMOG) readability score of 5 across the treatment rooms; these scores correspond to 5th-6th grade reading levels.

### Program Structure and CBT Modules

**Fig. 3:**
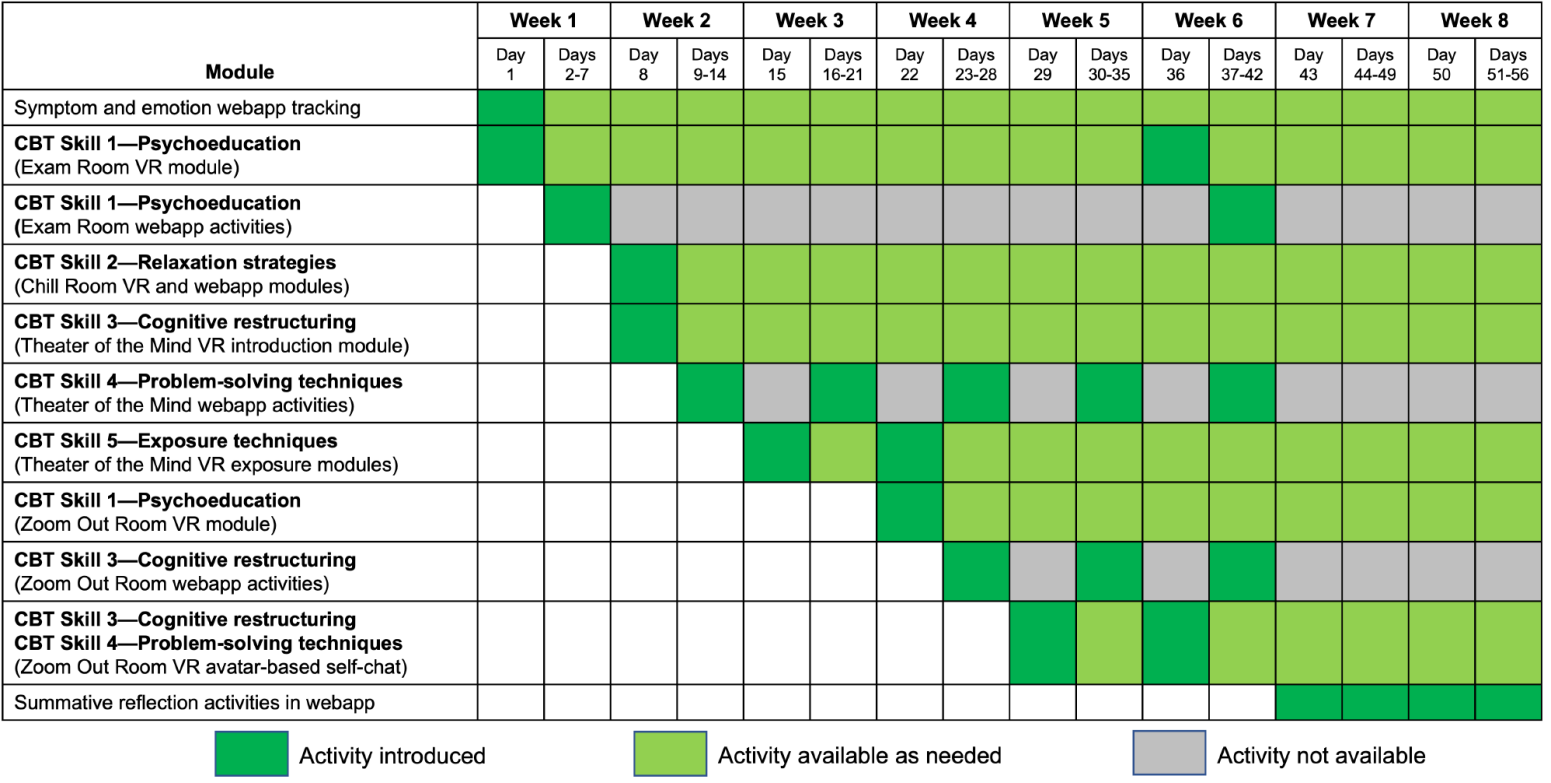
8-week CBT treatment protocol as delivered in VR. See text for details

The standardized CBT program is self-administered and includes a network of environments arranged in a virtual clinic that participants experience at home though a protocolized, 8-week program. Figure 3 presents the activities for each day. Depending on the module, daily treatments last between 5 and 20 minutes. The VR modules are reinforced with daily messages and CBT exercises delivered by a webapp that is available on any smartphone or computer along with emails. Messages have been programmed in a way to reinforce cognitive restructuring. The program progressively builds new skills and culminates in transitioning from using VR to applying the skills learned in VR to everyday life:

#### CBT Skill 1—Psychoeducation

In the *Exam Room*, participants virtually embody the role of a doctor to examine a patient with pain and discover anatomical and functional aspects of the GI tract. They learn about visceral hypersensitivity and engage in an immersive experience about the brain-gut axis. In the *Zoom Out Room*, participants journey through a series of “zoom out” maneuvers to gain perspective about the global community of people with IBS and, in the process, gain new perspective about themselves as a person with IBS. In the *Chill Room*, another therapeutic opportunity emerges from the combination of immersive VR environments with advances in conversational artificial intelligence (AI) via the Mobile Artificially Intelligent Ally (MAIA). The integration of AI within VR offers an unprecedented opportunity for patients to participate in talk therapy, which usually requires one-on-one sessions with a licensed therapist, a resource that is not always available or affordable for many people.

#### CBT Skill 2—Relaxation Strategies

In the *Chill Room*, which has been extensively used at Cedars-Sinai in over 200 participants in our inpatient VR consult service, participants practice biofeedback-enabled breath training, experience gut-directed hypnotherapy based on published studies using this technique for IBS, [17, 69–71] and learn to relax in nature environments that leverage the known therapeutic benefits of “biophilia,” which refers to the innate connection that humans have with natural environments, particularly green spaces [72–74]. Prior research has employed biophilic VR programs to reduce stress and support positive psychology [74, 75]. 

#### CBT Skill 3—Cognitive Restructuring

In the *Theater of the Mind*, participants learn principles of CBT, namely, that thoughts about gastrointestinal symptoms can be maladaptive and unhelpful and contribute to perceptions of gut pain, that such thoughts can be challenged and “restructured,” and that changing such thoughts can help alleviate gut pain and the negative emotions associated with it. Users enter a movie theater representing their own mind. Scenes on the screen depict thoughts as participants learn to identify or “catch” the thoughts associated with gut pain, to recognize when such thoughts contain distortions or errors, and to dispute such thoughts with challenging questions. Participants then learn to restructure such unhelpful thoughts about gut pain and replace them with thoughts that are more accurate, fact-based, balanced, and helpful, thereby leading to reductions in negative emotions and GI pain.

#### CBT Skill 4—Problem-Solving Techniques

In the *Theater of the Mind*, participants are provided with opportunities to practice applying the principles and techniques of CBT to challenge their perceptions of GI pain. In the *Zoom Out Room*, participants engage in avatar-based self-chat, through a validated technique called “virtual embodiment,” [76–84] to explore ways to manage their pain, combat the stigma often experienced by people with IBS, and learn to manage biopsychosocial distress through a process of self-discovery.

#### CBT Skill 5—Exposure Techniques

In the *Theater of the Mind Exposure Modules*, users practice CBT skills they learned in the earlier *Theater of the Mind* activities. Within the immersive VR environment, participants are visually “exposed” to the kind of stressful life situations associated with common DGBI symptoms that they might encounter in the real world. For example, for a patient with IBS, they are exposed to being in a public restroom with someone in the adjacent stall, experiencing pain while eating, or being in a meeting with other people while experiencing gut pain. Participants are prompted to practice and use the CBT skills they learned in earlier modules so that they can achieve a sense of mastery about how to handle such situations when they experience them in their lives.

### Initial Qualitative Assessment with IBS Patients

As part of Phase I (Design) in the ORBIT Model, we performed qualitative validation with 15 patients using the VR software [68]. Recruited patients met Rome IV criteria for IBS [1] and were receiving care at gastroenterology clinics at Cedars-Sinai. Patients who consented agreed to experience the VR and participate in debriefing sessions. Sessions were led by a qualitative research expert and discussions lasted 2 hours and were audiotaped and transcribed. Patients answered open-ended “think aloud” questions along with scripted probes. The questions focused on global perceptions, module length, instructional clarity, graphics quality, perceived effectiveness, and opportunities for improvement. Afterwards, we used an inductive thematic analysis in which debriefing transcripts were coded and labels identified within the unstructured data. After sorting, combining, and refining the codes and labels, inductive themes were defined and justified with verbatim quotes. Thematic saturation was achieved after interviews with 15 patients. The average age was 47 years and they had IBS for a median duration of 10 years. Fourteen (93%) patients had no, a little bit, or some experience with VR. No patients reported VR-related vertigo (“cybersickness”). Table 1 presents sample user feedback for each treatment room. Across demographics, VR experience, and years with IBS, patients expressed positive perceptions about using VR for IBS; all 15 (100%) said they would recommend VR to other people with IBS. The full methods and results of this Phase I qualitative validation study are available at this 2022 citation published in the *American Journal of Gastroenterology* [68].

**Table 1:** Patient perceptions of VR treatment rooms in initial qualitative validation testing [68]

Room	CBT Components	IBS Patient Perceptions
Exam Room	• Psychoeducation	• Visuals helped “better understand the connection between the gut and the mind.” • Patients appreciated “the visualization of how the gut and the brain interact.” • The module helped people envision “what doctors talk about when explaining IBS.”
Chill Room	• Relaxation strategies	• The Chill Room was “powerful,” “calming,” “serene,” and “soothing.” • Helped patients relax as “breathing slowed down with the expansion and contraction of the mandala”; “It was very helpful for me…the graphics, the calmness….” • Meditation was “helpful in bringing anxiety down” and to “feel better in the long run.”
Theater of the Mind	• Cognitive restructuring • Problem-solving skills • Exposure techniques	• CBT content was “valuable,” “easy to understand,” and “relatable”; “it’s reassuring … seeing how accurate you have the mental process of somebody with IBS.” • Experience caused some to reassess their IBS-related cognitions: “I am reflecting on two things I’ve picked up; saying the thought out loud and replacing it.” • Put patients’ minds “at ease” and helped them feel “less alone with intrusive thoughts.” • Helped people “realize how destructive self-blame thoughts can be.”
Zoom Out Room	• Psychoeducation • Cognitive restructuring • Problem-solving skills	• The perspective-taking content was considered “straightforward” and “self-driven.” • Engaging in self-talk helped patients go “beyond themselves” and “be more self-compassionate.” • Hearing themselves talk led to self-reflection and reconsideration of their IBS cognitions. • Some patients felt less alone: “When you see from space that there’s a lot of people with IBS…it makes me feel like one of many.”

### Iterative Updates to VR Program After Initial Qualitative Validation

After completion of the initial 12 patient interviews, 23 software changes were made to enhance the program. For example, in the *Exam Room*, patients recommended additional education on the role that the heart and cardiovascular system may have in the Management of Irritable Bowel Syndromeathogenesis of IBS and visceral pain. The voiceover script was thus expanded to introduce an example of developing “stomach cramps” while exercising, and illustrated how gut hypoperfusion can cause visceral pain. For the *Chill Room*, some participants thought that the desert scene felt “desolate” and requested a water environment, leading us to develop a lake scene. As for *Theater of the Mind*, patients recommended “trigger scenes” to practice CBT in stressful situations, such as being in a public restroom, being in the middle of a meeting when IBS symptoms occur, or flying in an airplane and needing to bother people to access the restroom; such trigger Management of Irritable Bowel Syndrome the VR program. Finally, for the *Zoom Out Room*, patients suggested adding “conversation starter” topics to help initiate the self-chat feature. We thus added questions such as: “Do you ever feel embarrassed about having IBS?”; “What steps can you take to gain control over your IBS?” After making the changes, we conducted 3 more interviews, which confirmed thematic saturation.

## Conclusion

While IBS is very common and overwhelmingly impacts HRQOL, current pharmacologic treatments often fall short in delivering meaningful improvements in symptoms and are commonly limited by side effects. Robust evidence indicates that CBT can improve symptoms and HRQOL in IBS but is rarely used and often inaccessible to patients due to the paucity of CBT-trained clinicians. Hence, it is vital to develop, test, and validate novel behavioral strategies that are effective, durable, safe, inexpensive, and accessible.

Use of a home-based, standardized, VR CBT program, has potential to bridge this gap. We are currently in Phase II of the ORBIT Model and are planning pilot RCTs to gather preliminary data assessing VR’s efficacy and feasibility in IBS. Based on what we observe in these pilot studies from patients and clinicians, additional enhancements will be made to the VR software and its integration in clinical workflows before subjecting it to a definitive, adequately powered efficacy trial. Moreover, data from these studies can inform future research examining VR CBT treatments for other DGBIs (e.g., functional dyspepsia, functional heartburn), Crohn’s disease, and ulcerative colitis, among other GI conditions. Given the high prevalence of IBS and other GI conditions combined with the paucity of effective medical therapies and marked shortage of trained CBT practitioners, the development and validation of novel self-administered approaches are needed.

## Data Availability

No datasets were generated or analysed during the current study.
